# Bile Sensing: The Activation of *Vibrio parahaemolyticus* Virulence

**DOI:** 10.3389/fmicb.2017.00728

**Published:** 2017-04-21

**Authors:** Vengadesh Letchumanan, Kok-Gan Chan, Tahir M. Khan, Sarah I. Bukhari, Nurul-Syakima Ab Mutalib, Bey-Hing Goh, Learn-Han Lee

**Affiliations:** ^1^Division of Genetics and Molecular Biology, Institute of Biological Sciences, Faculty of Science, University of MalayaKuala Lumpur, Malaysia; ^2^Novel Bacteria and Drug Discovery Research Group, School of Pharmacy, Monash University MalaysiaSelangor, Malaysia; ^3^Department of Pharmacy, Abasyn UniversityPeshawar, Pakistan; ^4^Department of Pharmaceutics, College of Pharmacy, King Saud UniversityRiyadh, Saudi Arabia; ^5^UKM Medical Molecular Biology Institute, UKM Medical Centre, Universiti Kebangsaan MalaysiaKuala Lumpur, Malaysia; ^6^Center of Health Outcomes Research and Therapeutic Safety (Cohorts), School of Pharmaceutical Sciences, University of PhayaoPhayao, Thailand

**Keywords:** bacteria, human gastrointestinal tract, bile, *Vibrio parahaemolyticus*, type III secretion system 2

## Abstract

Bacteria must develop resistance to various inhospitable conditions in order to survive in the human gastrointestinal tract. Bile, which is secreted by the liver, and plays an important role in food digestion also has antimicrobial properties and is able to disrupt cellular homeostasis. Paradoxically, although bile is one of the guts defenses, many studies have reported that bacteria such as *Vibrio parahaemolyticus* can sense bile and use its presence as an environmental cue to upregulate virulence genes during infection. This article aims to discuss how bile is detected by *V. parahaemolyticus* and its role in regulating type III secretion system 2 leading to human infection. This bile–bacteria interaction pathway gives us a clearer understanding of the biochemical and structural analysis of the bacterial receptors involved in mediating a response to bile salts which appear to be a significant environmental cue during initiation of an infection.

## Introduction

Humans have a complex digestive system that not only aids in digestion of food but also has a role in self-defense against microorganisms in the body. Microorganisms such as bacteria have to tolerate various extreme environments in order to survive in the human gastrointestinal tract (Begley et al., 2005). Bile is an alkaline substance that is continuously secreted by liver and stored in the gall bladder in humans; the presence of bile plays an important role in the digestive system process. During the digestive process, the lipids are emulsified and solubilized by bile. In addition, bile has the capability to affect the cell membranes proteins and phospholipid structures and cause cellular homeostasis. Bile aids in the emulsification and solubilization of lipids in the gastrointestinal tract. In addition, it has the capability to affect the phospholipids and proteins of cell membranes and disrupt cellular homeostasis. Hence the ability to overcome the potentially lethal effects of bile is important for bacteria in order to survive and subsequently colonize the gastrointestinal tract (Begley et al., 2005; Hung and Mekalanos, 2005; Edwards and Slater, 2009). Recently, there has been increased evidence showing bile is been used as a signaling cue by enteric bacteria to initiate virulence genes in host infection (Pope et al., 1995; Krukonis and DiRita, 2003; Prouty et al., 2004). Bile acids are a major component of crude bile that triggers the expression of bacterial virulence in the body. In this article, we aim to discuss how *Vibrio parahaemolyticus* senses bile in the human GI tract to regulate type III secretion system 2. This bile–bacteria interaction pathway gives us a clearer understanding of the biochemical and structural analysis of the bacterial receptors that takes action upon sensing the bile salts during an infection.

## Virulence Factors of *Vibrio parahaemolyticus*

The bacterial protagonist in this story is *V. parahaemolyticus*, a Gram-negative, halophilic bacterium which naturally inhabits marine and estuarine environments worldwide (Zhang and Orth, 2013; Letchumanan et al., 2014). *V. parahaemolyticus* is recognized as the causative agent of foodborne gastroenteritis, a disease often associated with consumption of raw or undercooked seafood (Raghunath, 2015). Global climate change and rising ocean temperatures have led to the increase in the distribution of this pathogen worldwide (O’Boyle and Boyd, 2014). This is of concern as approximately half the reported foodborne cases in Asian countries are caused by *V. parahaemolyticus* (Alam et al., 2002; Bhuiyan et al., 2002). Frequent outbreaks of *V. parahaemolyticus* cases have also been reported in the United States and coastal countries of Europe such as Spain, Italy, and Norway (Caburlotto et al., 2008; Scallan et al., 2011; Ottaviani et al., 2013).

*V. parahaemolyticus* possess wide range of virulence factors that enables them to cause a gastrointestinal infection including adhesin (Liu and Chen, 2015), toxins, and secreted effectors (Zhang and Orth, 2013). These virulence factors play a vital role in the pathogenesis of the disease. During the initial host cell binding, adhesion is the first important step in bacterial pathogenesis (Liu and Chen, 2015). This factor is present on the surface of all *V. parahaemolyticus* to form a platform for them to attach onto host cell and secrete toxins during an infection (Broberg et al., 2011; Zhang and Orth, 2013; Letchumanan et al., 2014). The thermostable direct hemolysin (*tdh*) and TDH related hemolysin (*trh*) are the two major toxins found in *V. parahaemolyticus* (Honda et al., 1988; Nishibuchi et al., 1992; Okada et al., 2009). These two virulence toxins are believed to cause hemolysis and cytotoxic activity in a host cell (Broberg et al., 2011; Ceccarelli et al., 2013). The *tdh* is a pore-forming toxin which forms pores in the erythrocyte’s membrane (Matsuda et al., 2010). The large pore size enables both water and ions to flow through the membrane (Honda and Iida, 1993). The subsequent alterations in ion flux in the intestine causes the diarrhea which is observed during an infection (Raghunath, 2015). Similar to the *tdh* gene, the *trh* gene also triggers cl^-^ channels resulting in altered ion flux during an infection (Takahashi et al., 2000). Both the *tdh* and *trh* are correlated with pathogenic *V. parahaemolyticus* strains, however, these genes do not completely account for the pathogenicity of *V. parahaemolyticus* (Lynch et al., 2005). There are several studies have reported that even in the absence of *tdh* and/or *trh* genes, *V. parahaemolyticus* strains remain virulent indicating the existence of other virulence factors (Jones et al., 2012; Pazhani et al., 2014). The thermolabile hemolysin (*tlh*), a type of phospholipase is another virulence toxin found in *V. parahaemlyticus* (DePaola et al., 2003; Zhang and Austin, 2005). Although the specific function of this gene in human infection remains unclear, *tlh* gene expression is upregulated under conditions mimic the intestinal environmental of human (Broberg et al., 2011; West et al., 2013). Hence, in the process of infection, *tlh* gene may be equally important as the *tdh* and *trh* genes.

The type III secretion system (T3SSs) is another important virulence factor of *V. parahaemolyticus* which is responsible for its pathogenicity (Broberg et al., 2011). This protein like structure has a secretion apparatus consisting of three main parts: the basal body that extends into the inner and outer membranes; a needle like structure that allows toxins to travel; and the translocon which is a pore injected into a target cell membrane (Izore et al., 2011). The T3SS1 and T3SS2 are the two main T3SSs encoded by *V. parahaemolyticus*. The cytotoxic T3SS1 is reported to be present in all *V. parahaemolyticus* and causes mouse lethality and possible initiation of autophagy (Park et al., 2004; Burdette et al., 2009; Hiyoshi et al., 2010). The enterotoxin T3SS2, on the other hand plays a vital part in determining the environmental fitness of strains (Hiyoshi et al., 2010; Matz et al., 2011). The T3SS2, *tdh* and *trh* are also known to be encoded on the pathogenicity island (Vp-PAI), signifying that *V. parahaemolyticus* acquires virulence determines through horizontal gene transfer (Okada et al., 2009; Matz et al., 2011). It is believed that the progression and severity of infection in humans are effected by the *V. parahaemolyticus* T3SS toxins (Ono et al., 2006). The strains that possess this needle-like T3SSs have the advantage of being able to secrete bacterial protein effectors directly into the host cell membrane and cytoplasm without facing the extracellular environment (Cornelis, 2006). In addition, the T3SS2 is suggested to be associated with *tdh*- and/or *trh*-positive *V. parahaemolyticus* strains (Raghunath, 2015). There are two distinct lineages of T3SS2 that have been described and associations were demonstrated of *tdh* with T3SSα and *trh* with T3SSβ (Park et al., 2004; Noriea et al., 2010). This could suggest that *V. parahaemolyticus* strains with the *tdh* and/or *trh* genes and T3SSs system have better ability to overcome host defenses in humans, conferring virulence that facilitates the development of infection.

Further analysis on the virulence properties has led to the discovery of type VI secretion systems encoded by T6SS1 and T6SS2 in *V. parahaemolyticus*. The T6SS1 is located on chromosome 1 where else, T6SS2 is located on chromosome 2 on *V. parahaemolyticus* RIMD 2210633 (Boyd et al., 2008; Izutsu et al., 2008). Salomon et al. (2013) proposed the role of T6SSs in *V. parahaemolyticus*. The T6SS1 is very active under warm marine-like conditions where else, T6SS2 is active under low salt conditions. It is also noted that surface sensing and quorum sensing differentially regulate both systems (Salomon et al., 2013). The T6SS2 and T3SS2 co-exist, suggesting the both systems may cooperate during an infection. T6SS2 takes the first step of infection as a role of adhesion where else T3SS2 exports effectors by inducing enterocytotoxicity (Park et al., 2004; Yu et al., 2012).

## The Sensing of Bile

*V. parahaemolyticus* with the virulence factors described are able to launch an attack on and cause illness to humans. Even with its arsenal of virulence factors, this bacterium still has to first survive the harsh conditions in the human gastrointestinal tract. It is suggested that exposure to harsh environmental conditions enables bacteria to be able to withstand the effects of bile in humans. The various pH conditions, temperatures and growth harden the bacteria toward the antimicrobial effects of bile in human. This will eventually increase their tolerance toward bile and the bacteria is able to survive in the human gastrointestinal tract. In addition, the bile levels in the human intestine are not constant and particularly in the presence of food, the bacteria would be less affected by the bile (Begley et al., 2005). Therefore, with these added advantages, it could be suggested that *V. parahaemolyticus* can indeed survive in the human gastrointestinal tract and regulate virulence during infections.

Bile is a bactericidal agent that are made up from various proteins, ions, pigments, cholesterol and bile salts. In an infection, the bile salts is believe to provide protection against bacteria (Merritt and Donaldson, 2009). When there is high amount of bile acids in the small intestine, the bacterial growth is inhibited (Inagaki et al., 2006). Where else, the growth of bacteria increases in the small intestine when bile is secreted in low amount, such seen in liver cirrhosis patients (Slocum et al., 1992). However, enteric pathogens including *Vibrio* species have now developed a mechanism to resist the action off bile.

Gotoh et al. (2010) discovered the production of T3SS2 proteins are induced by bile under osmotic conditions similarly to the environments in gastrointestinal tract. They identified that the T3SS2 system is encoded in the pathogenicity island (Vp-PAI) and causes enterotoxicity effects to host cell. The VtrA and VtrB are the two transcriptional regulators that regulates encoded genes. Based on the study, *V. parahaemolyticus* initially recognizes its location in the human gastrointestinal tract by detecting bile acids. The transcription of Vp-PAI will be induced by bile acids via two main proteins, the VtrA and VtrB. The virulence genes then are regulated by the transduction of signals in the human intestinal tract (Gotoh et al., 2010). It was revealed that crude bile is a potent host derived inducer of *tdh* gene and T3SS2 under osmotic conditions corresponding to those in the intestinal tract.

**FIGURE 1:**
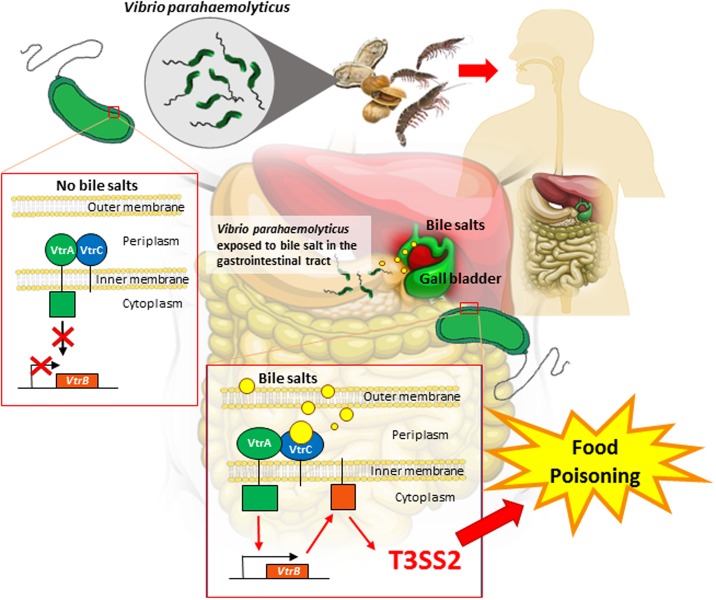
**Illustration on how *Vibrio parahaemolyticus* sense bile salts in the human gastrointestinal tract.** Human ingest *V. parahaemolyticus* from contaminated seafood such as clams, shrimps and oysters. Upon ingestion, *V. parahaemolyticus* is exposed to bile salts in the gastrointestinal tract. VtrA and VtrC are two gene encoded proteins that is identified to be responsible for bile salt sensing. VtrA and VtrC will interact and form a protein complex on the surface of the membrane of the bacterial cell. This complex structure then binds to bile salts and triggers the cell to produce toxins. Upon binding of bile salts to the VtrA/VtrC complex, the cytoplasmic DNA binding domain of VtrA is activated which in turn induces VtrB to activate, resulting in the T3SS2 expression. T3SS2 virulence is secreted thus causing illness to human.

Recently, Li et al. (2016) reported how *V. parahaemolyticus* has the ability to sense bile as an environmental cue to regulate its virulence mainly the T3SS2 during an infection. The study utilized bioinformatics tools to identify the proteins that are responsible for bile salt sensing and T3SS2 activation. **Figure 1** illustrates how the bacteria-bile sensing mechanism happens in the human body. VtrA and VtrC are two gene encoded proteins that is identified to be responsible for bile salt sensing. These two genes interact to form a protein complex on the surface of the membrane that surrounds the bacterial cell. The two proteins then create a barrel like structure that binds to bile salts and triggers the cell to produce toxins. Upon binding of bile salts to the hydrophobic chamber in the VtrA/VtrC complex, the cytoplasmic DNA binding domain of VtrA is activated which in turn induces VtrB to activate the T3SS2 virulence system. The VtrA/VtrC complex is described to be highly conserved in a group of diverse Vibrionaceae family (Li et al., 2016). Additionally, the study also found a family of monomeric lipid binding calycin domain proteins that has expanded to include an obligate heterodimer which binds to bile salts and can be utilized to transmit a signal. This increases the ability of *V. parahaemolyticus* to sense bile salt as an environmental cue to regulate virulence.

It is well understood that enteric bacteria including *V. parahaemolyticus* has the ability to sense bile which helps them identify their immediate environments and virulence factors can be expressed. *V. parahaemolyticus* releases toxins and type III secretion systems (T3SS2) in order to trigger virulence during an infection. This mode of mechanism ensures the survival of pathogenic *V. parahaemolyticus* in the environments and increase in the bacterial infections. However, this mechanism will cause more harm to we humans in future. Our own body defense fails to protect us against bacterial infections and on the other hand helps bacteria to release virulence. This situation will be worsened by the emergence of antimicrobial resistant strains in the environment which has become a major therapeutic challenge. As the effectiveness of treating bacterial infections declines, interest has been renewed toward using bacteriophages as a non-antibiotic approach to control the spreading of evolutionary *V. parahaemolyticus* strains worldwide (Wittebole et al., 2014; Letchumanan et al., 2016). Bacteriophage belonging to the *Siphoviridae* family is suitable in controlling *Vibrio* species (Letchumanan et al., 2016). This bacteriophage is highly specific to the bacterial host cell, do not affect or alter the gut microbiota (Hagens and Offerhaus, 2008), and safe to be consumed by humans. The phages are able to perform as a bio-control agent to control and inhibit virulence of pathogenic *Vibrio* species from clinical and environmental samples (Jassim and Limoges, 2014). In addition, the application of bacteriophage in the aquaculture industry can reduce the dependency of antibiotics and control the spreading of antimicrobial resistant bacteria in the environment (Letchumanan et al., 2016). The listed advantages make bacteriophage therapy a promising tool to control bacterial infections.

## Conclusion and Future Perspective

In summary, bile salts in human not only aid during digestion of food but possess antimicrobial activities as they have the ability to inhibit the survival of bacteria in the human gastrointestinal tract. However, certain conditions enable *V. parahaemolyticus* to develop resistance toward bile and eventually use bile as an environmental cue to regulate virulence. In order to treat infections, it is important to understand how *V. parahaemolyticus* senses bile salts and how this relates to their ability to regulate their virulence in the host during an infection. Given that there have been increasing numbers of multidrug resistant *Vibrio* strains from both clinical and environmental studies worldwide, drugs targeting suppression of bacterial virulence mechanisms should be designed instead of focusing on killing or inhibiting the growth of bacteria. Seen in this light, researchers will be able to design new drugs that may prevent the production of bacterial toxins and alleviate food poisoning symptoms. Future studies could focus on how other disease causing bacteria sense environmental cues to produce virulence during an infection.

## Author Contributions

VL performed the literature review and manuscript writing. KG-C, TK, SB, N-SAM, B-HG, and L-HL provided vital guidance and insight to the writing. The project was conceptualized by L-HL.

## Conflict of Interest Statement

The authors declare that the research was conducted in the absence of any commercial or financial relationships that could be construed as a potential conflict of interest.
